# Use of machine learning to identify risk factors for coronary artery disease

**DOI:** 10.1371/journal.pone.0284103

**Published:** 2023-04-14

**Authors:** Alexander A. Huang, Samuel Y. Huang

**Affiliations:** 1 Department of Statistics and Data Science, Cornell University, Ithaca, New York, United States of America; 2 Department of MD Education, Northwestern University Feinberg School of Medicine, Chicago, Illinois, United States of America; 3 Department of Internal Medicine, Virginia Commonwealth University School of Medicine, Richmond, Virginia, United States of America; The University of the West Indies, JAMAICA

## Abstract

Coronary artery disease (CAD) is the leading cause of death in both developed and developing nations. The objective of this study was to identify risk factors for coronary artery disease through machine-learning and assess this methodology. A retrospective, cross-sectional cohort study using the publicly available National Health and Nutrition Examination Survey (NHANES) was conducted in patients who completed the demographic, dietary, exercise, and mental health questionnaire and had laboratory and physical exam data. Univariate logistic models, with CAD as the outcome, were used to identify covariates that were associated with CAD. Covariates that had a p<0.0001 on univariate analysis were included within the final machine-learning model. The machine learning model XGBoost was used due to its prevalence within the literature as well as its increased predictive accuracy in healthcare prediction. Model covariates were ranked according to the Cover statistic to identify risk factors for CAD. Shapely Additive Explanations (SHAP) explanations were utilized to visualize the relationship between these potential risk factors and CAD. Of the 7,929 patients that met the inclusion criteria in this study, 4,055 (51%) were female, 2,874 (49%) were male. The mean age was 49.2 (SD = 18.4), with 2,885 (36%) White patients, 2,144 (27%) Black patients, 1,639 (21%) Hispanic patients, and 1,261 (16%) patients of other race. A total of 338 (4.5%) of patients had coronary artery disease. These were fitted into the XGBoost model and an AUROC = 0.89, Sensitivity = 0.85, Specificity = 0.87 were observed (Fig 1). The top four highest ranked features by cover, a measure of the percentage contribution of the covariate to the overall model prediction, were age (Cover = 21.1%), Platelet count (Cover = 5.1%), family history of heart disease (Cover = 4.8%), and Total Cholesterol (Cover = 4.1%). Machine learning models can effectively predict coronary artery disease using demographic, laboratory, physical exam, and lifestyle covariates and identify key risk factors.

## Introduction

Coronary artery disease (CAD) is the leading cause of death in both developed and developing nations [1]. CAD is an atherosclerotic disease that is associated with major complications, including angina, myocardial infarction, and sudden cardiac death [2–5]. Due to the high prevalence, morbidity, and mortality of CAD, identification of risk factors is a public health priority [6]. Genome-wide association studies have identified several genetic variants linked to CAD [7–10]. Additionally, epidemiological studies have identified significant socioeconomic, race, and sex disparities in CAD prevalence, quality measures, and outcomes [1, 11–13]. Further work has found that a combination of genetic, demographic, and environmental factors contributes to the severity of CAD and other cardiovascular diseases [1, 14–17]. Furthermore, lifestyle factors, such as diet and exercise, have been found to play an important role in the risk for CAD and other cardiovascular diseases [6, 18–20]. These studies have been combined to develop joint risk scores, factoring in both physiological covariates (blood pressure, cholesterol) as well as demographic covariates (age, race, gender) [5, 8, 9, 21]. Despite the strong literature studying the risk factors for CAD, most studies focus upon hypothesis testing or epidemiology focusing upon specific risk factors of interest [22–24]. While CAD is recognized as being of “multifactorial” cause, little is known regarding the relative predictive power of different risk factors (lifestyle vs genetic vs chronic disease comorbidities). Given these limitations in the literature, we will leverage transparent machine-learning methods including Shapely Additive Explanations (SHAP model explanations) and model gain statistics to identify pertinent risk-factors for CAD and compute their relative contribution to model prediction of CAD risk; the NHANES 2017–2020 cohort, a large, nationally representative sample of US adults, will be used within this study.

## Methods

A retrospective, cross-sectional cohort study using the publicly available National Health and Nutrition Examination Survey (NHANES) was conducted in patients who completed the demographic, dietary, exercise, and mental health questionnaire and had laboratory and physical exam data.

### Ethics approval and consent to participate

The acquisition and analysis of the data within this study was approved by the National Center for Health Statistics Ethics Review Board.

### Dataset and cohort selection

The National Health and Nutrition Examination Survey (NHANES 2017–2020) is a program designed by the National Center for Health Statistics (NCHS), which has been leveraged to assess the health and nutritional status of the United States population [25]. The NHANES dataset is a series of cross-sectional, complex, multi-stage surveys conducted by the Centers for Disease Control and Prevention (CDC) on a nationally representative cohort of the United States population to provide health, nutritional, and physical activity data. In the present study, we analyzed adult (≥18 years old) patients in the NHANES dataset who completed the demographic, dietary, exercise, and mental health questionnaire and had laboratory and physical exam data.

### Assessment of coronary artery disease

The medical conditions file was used to define coronary artery disease. Participants were asked: “Has a doctor or other health professional ever told you that you have coronary heart disease?” Participants who answered “Yes” to this question were considered as having CAD within this study.

### Independent variable

Potential model covariates were identified within the demographics, dietary, physical examination, laboratory, and medical questionnaire datasets in NHANES. All covariates were extracted and merged with the CAD indicator.

### Model construction and statistical analysis

Univariate logistic models, with CAD as the outcome, were used to identify covariates that were associated with CAD. Covariates that had a p<0.0001 on univariate analysis were included within the final machine-learning model. The machine learning model XGBoost was used due to its prevalence within the literature as well as its increased predictive accuracy in healthcare prediction. XGBoost models were fit with a train:test (80:20), and model accuracy statistics (AUROC, Sensitivity, Specificity, F1, Balanced Accuracy) were computed. Model covariates were ranked according to the Gain, Cover, and Frequency (representations of the relative contribution (“model importance”) of each of the covariates) to identify risk factors for CAD. The Gain statistic represents the overall proportion of the model prediction is attributed to a given statistic. The Cover and Frequency are representations of the proportion of trees that each of the covariates appear within the machine-learning model. SHAP explanations were utilized to visualize the relationship between these potential risk factors and CAD.

## Results

Table 1 shows that f the 7,929 patients that met the inclusion criteria in this study, 4,055 (51%) were female, 2,874 (49%) were male. The mean age was 49.2 (SD = 18.4), with 2,885 (36%) White patients, 2,144 (27%) Black patients, 1,639 (21%) Hispanic patients, and 1,261 (16%) patients of another race. A total of 338 (4.5%) of patients had coronary artery disease.

**Table 1:** Demographic information.

Coronary Artery Disease Grouping	All Patients	Coronary Heart Disease	No Coronary Heart Disease	P-Values
Age; Mean (SD)	49.23 (18.35)	68.34 (10.49)	48.38 (18.16)	P<0.001
Gender Female; Count (Proportion)	4055 (0.51)	92 (0.27)	3963 (0.52)	0.19
Gender Male; Count (Proportion)	3874 (0.49)	246 (0.73)	3628 (0.48)	
Race White; Count (Proportion)	2885 (0.36)	210 (0.62)	2675 (0.35)	0.21
Race Black; Count (Proportion)	2144 (0.27)	47 (0.14)	2097 (0.28)	
Race Hispanic; Count (Proportion)	1639 (0.21)	48 (0.14)	1591 (0.21)	
Race Other; Count (Proportion)	1261 (0.16)	33 (0.1)	1228 (0.16)	
Income_Poverty_Ratio; Mean (SD)	2.6 (1.63)	2.68 (1.53)	2.6 (1.64)	P<0.001
Albumin, urine (mg/L); Mean (SD)	47.96 (272.25)	159 (695.36)	43.02 (235.41)	P<0.001
Creatinine, urine (mg/dL); Mean (SD)	132.8 (87.58)	115.54 (69.81)	133.56 (88.21)	P<0.001
Albumin creatinine ratio (mg/g); Mean (SD)	46.65 (296.14)	167.66 (773.04)	41.28 (253.85)	P<0.001
Direct HDL-Cholesterol (mg/dL); Mean (SD)	53.51 (16.03)	48.27 (13.22)	53.75 (16.1)	P<0.001
LDL-Cholesterol, Friedewald (mg/dL); Mean (SD)	107.67 (35.57)	88.52 (38.53)	108.53 (35.2)	P<0.001
Total Cholesterol (mg/dL); Mean (SD)	184.54 (41.08)	163.6 (42.63)	185.49 (40.76)	P<0.001
Lymphocyte percent (%); Mean (SD)	31.28 (8.96)	26.97 (9.08)	31.47 (8.9)	P<0.001
Monocyte percent (%); Mean (SD)	8.19 (2.23)	8.77 (2.3)	8.17 (2.22)	P<0.001
Segmented neutrophils percent (%); Mean (SD)	57.07 (9.66)	60.4 (9.42)	56.92 (9.65)	P<0.001
Eosinophils percent (%); Mean (SD)	2.78 (2.08)	3.16 (2.11)	2.77 (2.08)	P<0.001
Monocyte number (1000 cells/uL); Mean (SD)	0.58 (0.21)	0.63 (0.2)	0.57 (0.21)	P<0.001
Segmented neutrophils num (1000 cell/uL); Mean (SD)	4.18 (1.71)	4.5 (1.54)	4.17 (1.72)	P<0.001
Eosinophils number (1000 cells/uL); Mean (SD)	0.2 (0.17)	0.23 (0.16)	0.2 (0.17)	P<0.001
Mean cell volume (fL); Mean (SD)	88.4 (6.19)	89.93 (5.94)	88.33 (6.19)	P<0.001
Red cell distribution width (%); Mean (SD)	13.9 (1.39)	14.33 (1.59)	13.88 (1.37)	P<0.001
Platelet count (1000 cells/uL); Mean (SD)	246.35 (65.54)	212.2 (58.43)	247.87 (65.43)	P<0.001
RBC folate (ng/mL); Mean (SD)	514.51 (230.32)	676.1 (345.28)	509.04 (223.43)	P<0.001
Serum total folate (nmol/L); Mean (SD)	39.29 (27.09)	58.23 (46.94)	38.64 (25.89)	P<0.001
5-Methyl-tetrahydrofolate (nmol/L); Mean (SD)	36.46 (22.3)	53.15 (41.16)	35.88 (21.12)	P<0.001
Tetrahydrofolate (nmol/L); Mean (SD)	0.77 (0.57)	1.08 (0.8)	0.76 (0.56)	P<0.001
Mefox oxidation product (nmol/L); Mean (SD)	1.75 (1.91)	2.81 (2.27)	1.71 (1.88)	P<0.001
Glycohemoglobin (%); Mean (SD)	5.82 (1.09)	6.42 (1.32)	5.79 (1.08)	P<0.001
Blood lead (ug/dL); Mean (SD)	1.16 (1.16)	1.57 (1.31)	1.14 (1.15)	P<0.001
Fasting Glucose (mg/dL); Mean (SD)	112.57 (36.98)	131.06 (48.49)	111.75 (36.18)	P<0.001
Alkaline Phosphatase (ALP) (IU/L); Mean (SD)	78.23 (26.94)	85.84 (30.2)	77.89 (26.73)	P<0.001
Blood Urea Nitrogen (mg/dL); Mean (SD)	14.86 (6.02)	20.06 (9.06)	14.63 (5.74)	P<0.001
Creatinine, refrigerated serum (mg/dL); Mean (SD)	0.91 (0.5)	1.15 (0.75)	0.89 (0.48)	P<0.001
Glucose, refrigerated serum (mg/dL); Mean (SD)	101.39 (35.15)	116.48 (42.96)	100.7 (34.6)	P<0.001
Lactate Dehydrogenase (LDH) (IU/L); Mean (SD)	158.13 (35.03)	166.62 (39.66)	157.74 (34.76)	P<0.001
Osmolality (mmol/Kg); Mean (SD)	281.34 (5.61)	284.06 (7.03)	281.21 (5.5)	P<0.001
Potassium (mmol/L); Mean (SD)	4.09 (0.36)	4.27 (0.43)	4.08 (0.35)	P<0.001
Total Bilirubin (mg/dL); Mean (SD)	0.46 (0.28)	0.52 (0.3)	0.45 (0.28)	P<0.001
Cholesterol, refrigerated serum (mg/dL); Mean (SD)	184.87 (41.12)	164 (42.45)	185.81 (40.8)	P<0.001
Total Protein (g/dL); Mean (SD)	7.15 (0.45)	7.02 (0.46)	7.16 (0.45)	P<0.001
Uric acid (mg/dL); Mean (SD)	5.4 (1.47)	5.93 (1.5)	5.37 (1.47)	P<0.001
BMXWT—Weight (kg); Mean (SD)	84.02 (23.31)	88.16 (22.7)	83.83 (23.33)	P<0.001
BMXWAIST—Waist Circumference (cm); Mean (SD)	100.65 (17.47)	108.3 (14.99)	100.33 (17.5)	P<0.001
SMQ020—Smoked at least 100 cigarettes in life; Mean (SD)	3261 (0.41)	208 (0.62)	3053 (0.4)	P<0.001
SMQ681—Smoked tobacco last 5 days? 1; Mean (SD)	1722 (0.22)	49 (0.14)	1673 (0.22)	P<0.001
MCQ053—Taking treatment for anemia/past 3 mos 1; Mean (SD)	412 (0.05)	33 (0.1)	379 (0.05)	P<0.001
MCQ080—Doctor ever said you were overweight 1; Mean (SD)	3175 (0.4)	187 (0.55)	2988 (0.39)	P<0.001
MCQ092—Ever receive blood transfusion 1; Mean (SD)	879 (0.11)	90 (0.27)	789 (0.1)	P<0.001
MCQ160a—Doctor ever said you had arthritis 2; Mean (SD)	5188 (0.65)	136 (0.4)	5052 (0.67)	P<0.001
MCQ160a—Doctor ever said you had arthritis 1; Mean (SD)	2345 (0.3)	202 (0.6)	2143 (0.28)	P<0.001
MCQ160m—Ever told you had thyroid problem 1; Mean (SD)	908 (0.11)	77 (0.23)	831 (0.11)	P<0.001
MCQ160p—Ever told you had COPD, emphysema, ChB 1; Mean (SD)	717 (0.09)	101 (0.3)	616 (0.08)	P<0.001
MCQ520—Abdominal pain during past 12 months? 1; Mean (SD)	1693 (0.21)	120 (0.36)	1573 (0.21)	P<0.001
MCQ300c—Close relative had diabetes? 1; Mean (SD)	3653 (0.46)	211 (0.62)	3442 (0.45)	P<0.001
MCQ300a—Close relative had heart attack? 1; Mean (SD)	1011 (0.13)	109 (0.32)	902 (0.12)	P<0.001
MCQ366a—Doctor told you to control/lose weight 1; Mean (SD)	2275 (0.29)	151 (0.45)	2124 (0.28)	P<0.001
PHQ_9; Mean (SD)	3.18 (4.3)	4.2 (5.75)	3.13 (4.22)	P<0.001
Caffeine..mg..1; Mean (SD)	137.22 (202.64)	183.64 (269.28)	135.16 (198.93)	P<0.001
Median.liver.stiffness; Mean (SD)	6.05 (5.26)	7.52 (8.3)	5.99 (5.09)	P<0.001
Direct.HDL.Cholesterol..mg.dL.; Mean (SD)	53.51 (16.03)	48.27 (13.22)	53.75 (16.1)	P<0.001
LBDLDL…LDL.Cholesterol..Friedewald..mg.dL.; Mean (SD)	107.67 (35.57)	88.52 (38.53)	108.53 (35.2)	P<0.001
Systolic_1; Mean (SD)	124.09 (19.51)	131.52 (23.28)	123.76 (19.26)	P<0.001
Diastolic_1; Mean (SD)	74.85 (11.76)	71.46 (12.71)	75 (11.7)	P<0.001

Descriptive statistics for demographic characteristics and all covariates within the machine learning model, stratified by whether patients had coronary artery disease.

**Fig 1:**
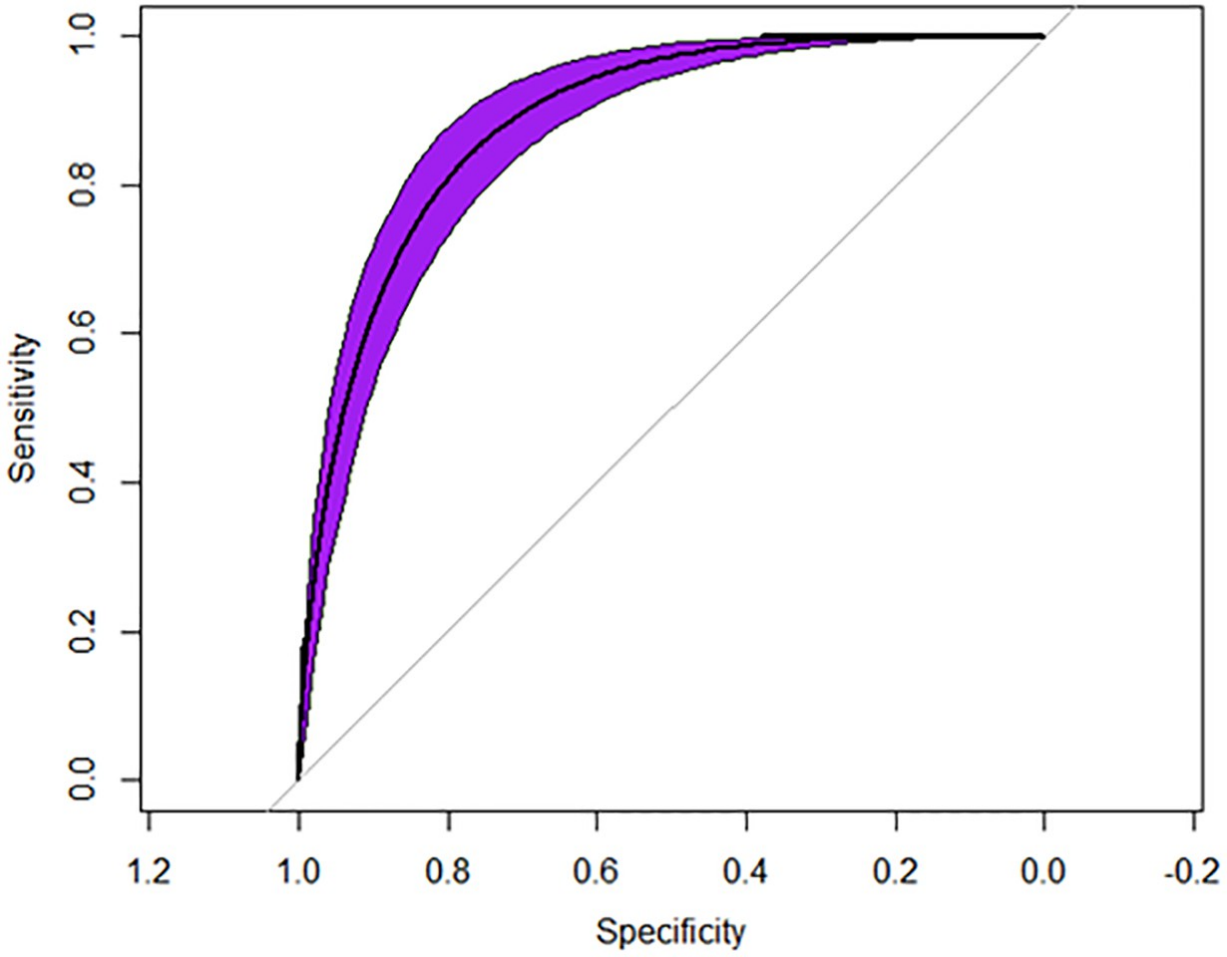
Restricted operator characteristic curve and model statistics. The ROC Curve for the machine-learning model predicting coronary artery disease. AUROC = 0.89.

The machine learning model had 58 out of a total 684 features that were found to be significant on univariate analysis (P<0.0001 used). These were fitted into the XGBoost model and an AUROC = 0.89, Sensitivity = 0.85, Specificity = 0.87 were observed Fig 1.

Table 2 shows the top five highest ranked features by cover, a measure of the percentage contribution of the covariate to the overall model prediction, were age (Cover = 21.1%), Platelet count (Cover = 5.1%), family history of heart disease (Cover = 4.8%), and Total Cholesterol (Cover = 4.1%).

**Table 2:** Model feature importance statistics.

Feature	Gain	Cover	Frequency
Age	19.8%	22.0%	5.5%
Platelet count (1000 cells/uL)	4.7%	3.7%	4.3%
Albumin creatinine ratio (mg/g)	3.9%	4.8%	3.9%
Total Cholesterol (mg/dL)	3.6%	3.4%	3.2%
MCQ300a—Close relative had heart attack?	3.4%	6.3%	2.7%
Median.liver.stiffness	3.0%	3.5%	3.9%
Diastolic_1	2.9%	2.0%	3.4%
Blood Urea Nitrogen (mg/dL)	2.5%	3.9%	2.1%
Caffeine..mg..1	2.3%	2.0%	2.9%
Blood lead (ug/dL)	2.3%	1.7%	3.2%
Segmented neutrophils num (1000 cell/uL)	2.3%	1.5%	2.3%
Â Potassium (mmol/L)	2.2%	2.6%	3.0%
Cholesterol, refrigerated serum (mg/dL)	2.1%	1.7%	1.7%
Gender	2.0%	2.1%	1.3%
Â Direct HDL-Cholesterol (mg/dL)	1.8%	1.3%	2.0%
Lymphocyte percent (%)	1.8%	1.3%	2.1%
MCQ160p—Ever told you had COPD, emphysema, ChB	1.8%	1.4%	1.2%
BMXWT—Weight (kg)	1.8%	3.4%	3.4%
Systolic_1	1.7%	1.0%	2.4%
Mefox oxidation product (nmol/L)	1.6%	4.3%	2.8%
BMXWAIST—Waist Circumference (cm)	1.6%	1.6%	2.3%
Albumin, urine (mg/L)	1.6%	2.0%	2.5%
PHQ_9	1.6%	1.6%	1.7%
Eosinophils percent (%)	1.6%	0.8%	1.9%
Â Glycohemoglobin (%)	1.6%	2.2%	1.7%
Segmented neutrophils percent (%)	1.5%	0.8%	1.7%
Creatinine, urine (mg/dL)	1.5%	0.5%	2.1%
Â Alkaline Phosphatase (ALP) (IU/L)	1.5%	1.0%	2.1%
Lactate Dehydrogenase (LDH) (IU/L)	1.5%	0.4%	2.3%
LDL-Cholesterol, Friedewald (mg/dL)	1.4%	1.7%	1.9%
Red cell distribution width (%)	1.3%	1.0%	1.8%
Creatinine, refrigerated serum (mg/dL)	1.1%	2.5%	1.6%
Osmolality (mmol/Kg)	1.0%	0.9%	1.3%
Uric acid (mg/dL)	1.0%	0.4%	1.3%
RBC folate (ng/mL)	1.0%	0.9%	1.5%
Â Serum total folate (nmol/L)	0.9%	0.4%	1.0%
Tetrahydrofolate (nmol/L)	0.8%	0.3%	1.2%
MCQ520—Abdominal pain during past 12 months?	0.8%	0.6%	0.7%
Â Glucose, refrigerated serum (mg/dL)	0.8%	0.4%	1.2%
Mean cell volume (fL)	0.8%	0.8%	1.8%
Total Protein (g/dL)	0.8%	1.3%	1.2%
MCQ092—Ever receive blood transfusion	0.7%	0.6%	0.7%
Monocyte percent (%)	0.7%	0.2%	1.1%
MCQ300c—Close relative had diabetes?	0.7%	0.4%	0.5%
5-Methyl-tetrahydrofolate (nmol/L)	0.6%	0.3%	0.7%
Fasting Glucose (mg/dL)	0.6%	0.2%	0.9%
Monocyte number (1000 cells/uL)	0.6%	0.2%	0.6%
Â Total Bilirubin (mg/dL)	0.6%	0.3%	0.9%
MCQ366a—Doctor told you to control/lose weight	0.5%	0.6%	0.5%
MCQ080—Doctor ever said you were overweight	0.5%	0.4%	0.4%
SMQ020—Smoked at least 100 cigarettes in life	0.2%	0.2%	0.3%
MCQ160a—Doctor ever said you had arthritis	0.2%	0.2%	0.3%
MCQ160m—Ever told you had thyroid problem	0.2%	0.1%	0.3%
SMQ681—Smoked tobacco last 5 days?	0.1%	0.2%	0.4%
Eosinophils number (1000 cells/uL)	0.1%	0.2%	0.2%
MCQ053—Taking treatment for anemia/past 3 mos	0.1%	0.1%	0.1%

The Cover, Gain, and Frequency of all covariates within the XGBoost model.

**Fig 2:**
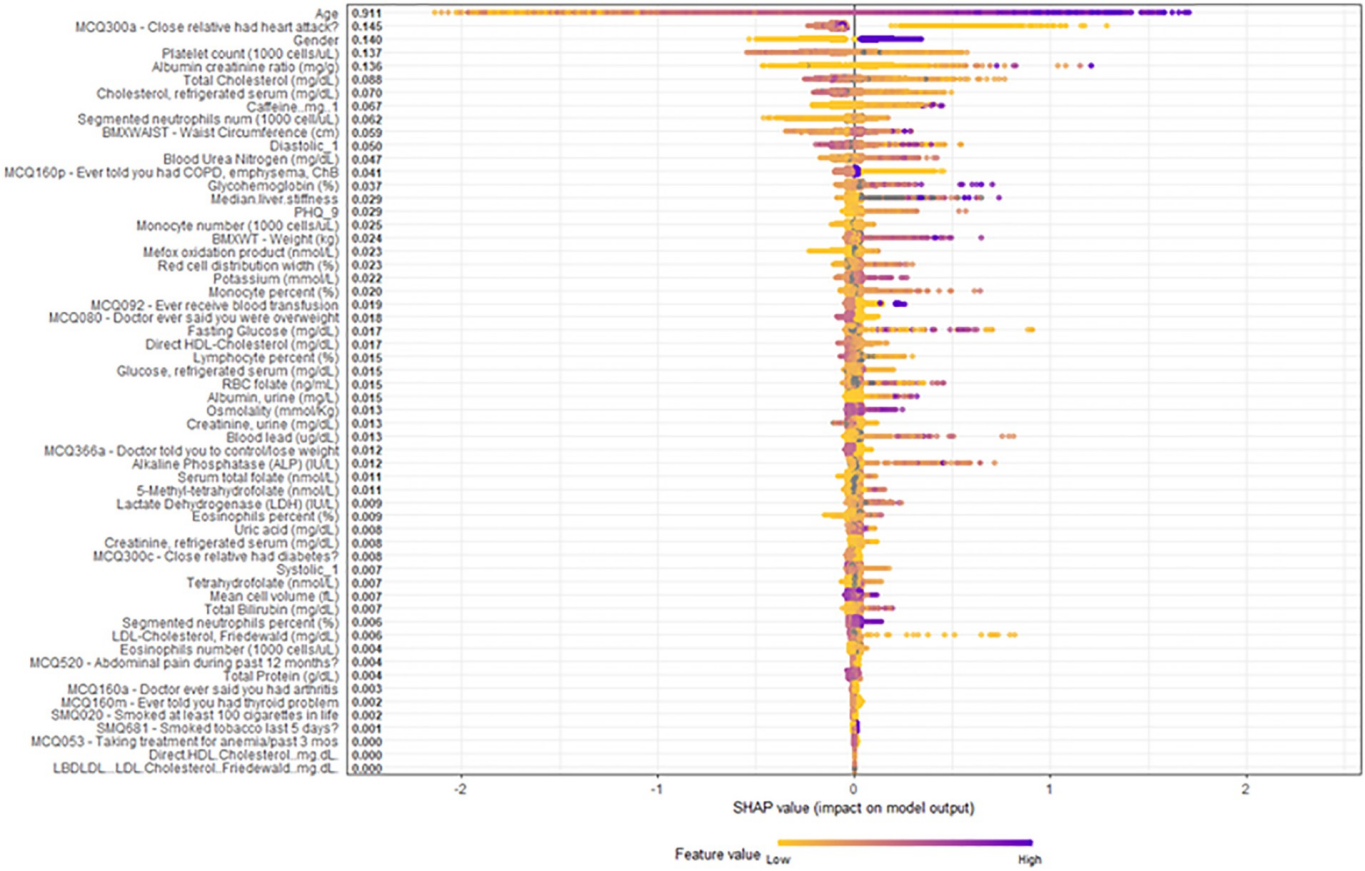
Overall SHAP explanations. SHAP explanations, purple color representing higher values of the covariate while yellow representing lower values of the covariate. X-axis is the change in log-odds for CAD.

In Fig 2, on SHAP visualization, we observed that: interpret the top four covariates age had a sigmoidal relationship with risk for coronary artery disease.

**Fig 3:**
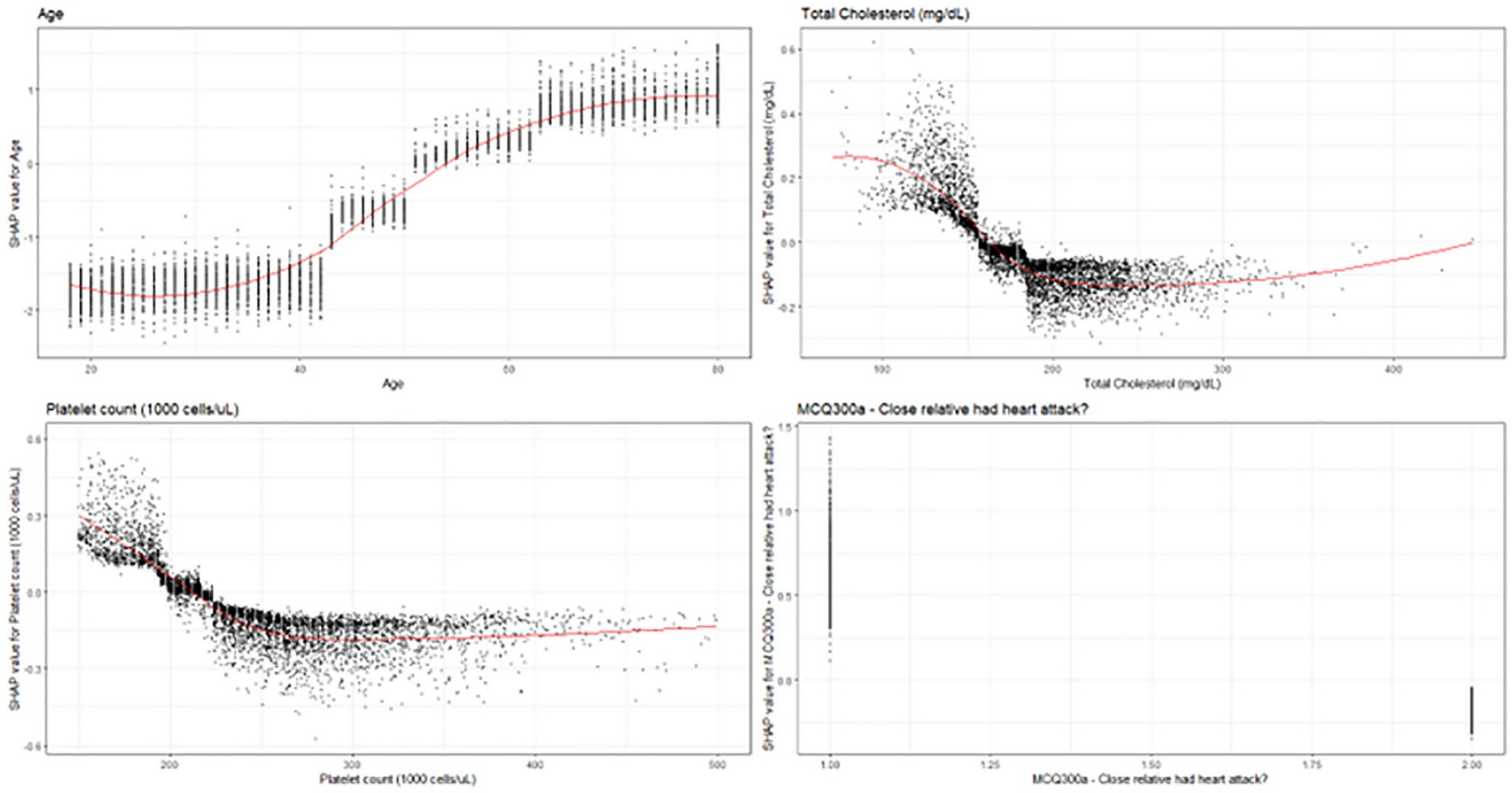
SHAP explanations for the top 4 covariates. SHAP explanations, covariate value on the x-axis, change in log-odds on the y-axis, red line represents the relationship between the covariate and log-odds for CAD, each black dot represents an observation. Covariates (top left—Age, top right—Total Cholesterol, bottom left—platelets, bottom right—Close relative with a heart attack (Yes = 1, no = 2)).

**Fig 4:**
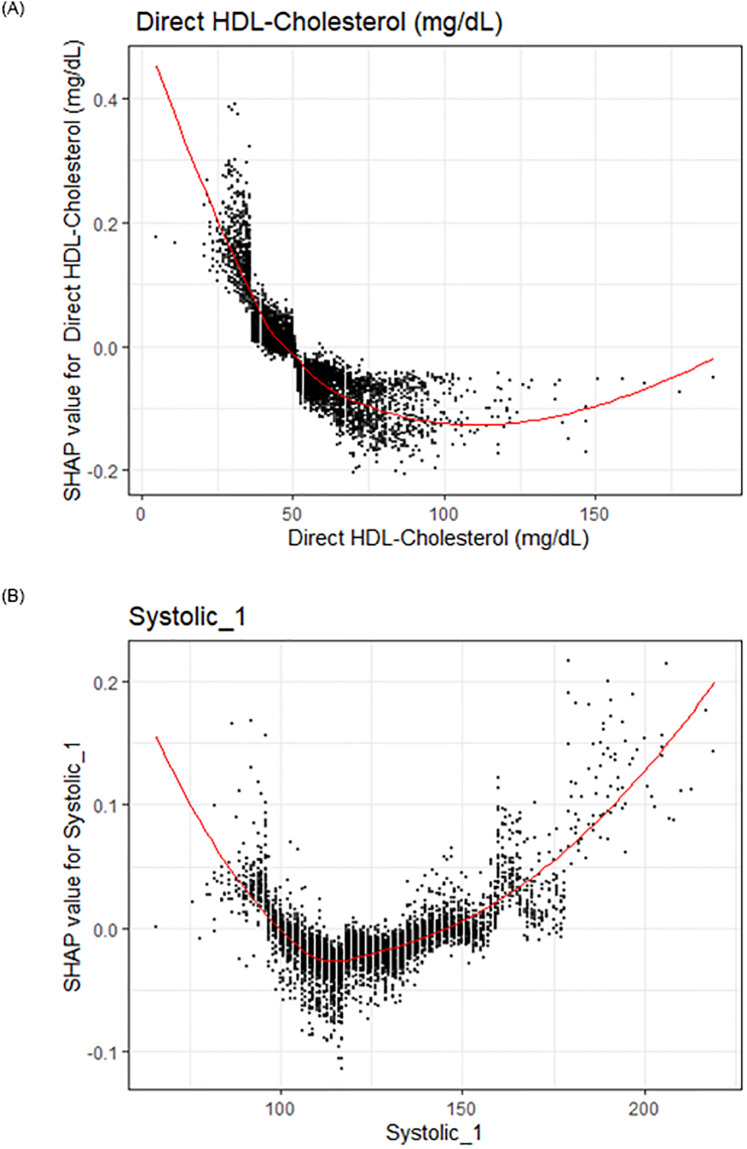
**a**: Covariates of interest to evaluate sensibility of the model. SHAP explanations for the relationship between HDL-Cholesterol and odds of CAD. Covariate value on the x-axis, change in log-odds on the y-axis, red line represents the relationship between the covariate and log-odds for CAD, each black dot represents an observation. **b**: SHAP explanations for the relationship between Systolic blood pressure and odds of CAD. Covariate value on the x-axis, change in log-odds on the y-axis, red line represents the relationship between the covariate and log-odds for CAD, each black dot represents an observation.

Figs 3, 4a and 4b shows the SHAP Explanations for various SHAP features. We observed that at ages between 20 and 35, there was no significant change in risk for CAD with increasing age, with age increasing between 35 and 70, there was a significant increase in risk for CAD with increasing age, and above 70 years of age, there was no significant increase in CAD with increasing age. Additionally, a curvilinear relationship was observed analyzing the relationship with total-cholesterol and risk for CAD. Patients with significantly decreased total cholesterol were observed to have increased risk for heart disease, and patients with increased cholesterol were observed to also be at increased risk, with a minimum risk around 200 mg/dL of cholesterol. A curvilinear relationship was also observed for the relationship between platelet count and risk for CAD, with significantly decreased platelet counts linked with CAD and significantly increased platelet counts also linked with CAD, a minimum observed around 300,000 cells/uL. Family history was also a significant predictor for CAD. Patients with close relatives having a heart attack in the past had significant increased risk for CAD.

## Discussion

In this retrospective, cross sectional cohort of United States adults, a machine learning model utilizing demographic, laboratory, physical examination, and lifestyle questionnaire data had strong predictive accuracy (AUROC = 0.89). The greatest predictors for coronary artery disease included age, total cholesterol, total platelets, and family history of a heart attack.

The visualizations completed for the top four covariates were concordant with current literature around the relationship between these covariates and coronary artery disease: there is strong epidemiological and physiological evidence for the link between increased age and cholesterol as major risk factors for coronary artery disease [26–28]. The non-linear relationship between cholesterol and coronary artery disease matches survival-modeling and restricted cubic spline analysis from other studies [28–36]. Furthermore, multiple genetic and sociological studies have found that family history is a significant risk factor for coronary artery disease [37–40]. Additionally, low-platelets being associated with coronary artery disease is associated with pathology such as thrombocytopenia [41–43]. In addition to the top four covariates within our model, we also wanted to explore if the machine-learning model was able to generate predictions for HDL-Cholesterol and Systolic Blood pressure, two major risk factors that have been widely studied within the cardiovascular literature. In these visualizations, we observed a strong negative relationship between HDL-cholesterol and risk for coronary artery disease (Fig 4a). We observe a curvilinear relationship between systolic blood pressure and coronary heart disease, with blood pressures lower than 120 being associated with increased risk for coronary heart disease and blood pressures above being strongly associated with coronary heart disease as well.

Since visualizations for risk factors match literature relationships, we have increased confidence that the machine learning model is able to capture the actual physiological relationships of these covariates [44–46]. These transparent machine-learning tools allow for increased confidence that these algorithms are picking up true signal within these covariates to predict coronary artery disease rather than just replicating potential biases stemming from systemic data- = quality errors that are present within the dataset. Additionally, these SHAP visualizations allow us to interpret that the increase predictive power of these machine-learning methods is associated with the ability for these non-parametric methods to more accurately capture the non-linear interactive relationship between the covariates, rather than just over-fitting the model to get increased accuracy.

The greatest strength of this algorithmic method for identification of the covariates is the ability to search through hundreds of covariates systematically without relying upon judgment form the researcher, which may be muddled by potential personal biases. This method also allows for the ranking of the relative importance of each of these covariates through the cover statistic, which allows us to obtain the relative contribution to the prediction each covariate has and thus infer from there an estimate for the relative contribution to true risk for coronary artery disease that each patient has. Another strength is that after these covariates are selected and the model built, SHAP visualizations can be used to make sure that each of the covariate either matches current literature understandings of the covariate’s association with coronary heart disease or in the case of a discrepancy, allow researchers to validate the plausibility of this feature and then evaluate for potential errors in data-quality.

Some potential weaknesses to this machine-learning analysis is the necessity of the retrospective nature of this cohort. The covariates that were selected within this study will be better at predicting coronary heart disease risk for this cohort than for other cohorts. However, this was limited by the use of training: testing sets to be able to minimize the errors that come with overfitting. Furthermore, visualizations of SHAP allow researchers to test for physiologic plausibility of each of these covariates and allows for effective analysis by researchers of whether these effects are due to true signal or if they are just noise that may be contributing to a type-1 error.

Given the analysis of the strengths and weaknesses of these methods, we argue that use of machine-learning methods can be an effective first step in the identification of risk-factors that can then be further selected by clinicians based upon the specific clinical presentation.

### Limitations

This study has several strengths and weaknesses. We utilized the NHANES dataset, which is a retrospective cohort, carrying the limitations of retrospective studies. However, this study allows for the selection of a large cohort, evaluation of data quality, and due to the publicly available nature of the cohort, allows for increased replication and follow-up studies based upon the same cohort. Furthermore, the cohort relied on surveys to obtain the outcome of interest (CAD) as well as the dietary and lifestyle information. More accurate measurements may have been achieved with prospective studies with automated measurement of foods. However, self-reported survey information allows for the volume of participants to be included within this study. Another weakness was the voluntary nature of this cohort, with participants choosing to opt into the study instead of being randomly selected. This may artificially select a different cohort that may significantly differ from the population. However, our analysis found a demographically diverse population, so these results may still be generalizable to other cohorts.

## Conclusion

Machine learning models can effectively predict coronary artery disease using demographic, laboratory, physical exam, and lifestyle covariates. Age, total cholesterol, total platelets, and family history of heart attack are the strongest predictors of coronary artery disease.

## References

[1] Albar HM, Alahmdi RA, Almedimigh AA, et al. Prevalence of coronary artery disease and its risk factors in Majmaah City, Kingdom of Saudi Arabia. *Front Cardiovasc Med*. 2022;9:943611. doi: 10.3389/fcvm.2022.943611 36158800 PMC9492946

[2] AlOthman AF, Sait ARW, Alhussain TA. Detecting Coronary Artery Disease from Computed Tomography Images Using a Deep Learning Technique. *Diagnostics (Basel)*. Aug 26 2022;12(9) doi: 10.3390/diagnostics12092073 36140475 PMC9498285

[3] Bhattad PB, Sherif AA, Mishra AK, Roumia M. Left Main Coronary Artery Disease: The Forgotten Lead of Electrocardiogram Is Predictive. *Cureus*. Aug 2022;14(8):e28391. doi: 10.7759/cureus.28391 36168367 PMC9506673

[4] Luu JM, Wei J, Shufelt CL, et al. Clinical Practice Variations in the Management of Ischemia With No Obstructive Coronary Artery Disease. *J Am Heart Assoc*. Sep 29 2022:e022573. doi: 10.1161/JAHA.121.022573 36172938 PMC9673699

[5] Maamari DJ, Brockman DG, Aragam K, et al. Clinical Implementation of Combined Monogenic and Polygenic Risk Disclosure for Coronary Artery Disease. *JACC Adv*. Aug 2022;1(3) doi: 10.1016/j.jacadv.2022.100068 36147540 PMC9491373

[6] de Souza ESCG, Buginga GC, de Souza ESEA, et al. Prediction of Mortality in Coronary Artery Disease: Role of Machine Learning and Maximal Exercise Capacity. *Mayo Clin Proc*. Aug 2022;97(8):1472–1482. doi: 10.1016/j.mayocp.2022.01.016 35431026

[7] Eurlings C, Bektas S, Sanders-van Wijk S, et al. Use of artificial intelligence to assess the risk of coronary artery disease without additional (non-invasive) testing: validation in a low-risk to intermediate-risk outpatient clinic cohort. *BMJ Open*. Sep 26 2022;12(9):e055170. doi: 10.1136/bmjopen-2021-055170 36167368 PMC9516207

[8] Gola D, Erdmann J, Muller-Myhsok B, Schunkert H, Konig IR. Polygenic risk scores outperform machine learning methods in predicting coronary artery disease status. *Genet Epidemiol*. Mar 2020;44(2):125–138. doi: 10.1002/gepi.22279 31922285

[9] Goodman MO, Cade BE, Shah N, et al. Pathway-Specific Polygenic Risk Scores Identify Obstructive Sleep Apnea-Related Pathways Differentially Moderating Genetic Susceptibility to Coronary Artery Disease. *Circ Genom Precis Med*. Sep 28 2022:101161CIRCGEN121003535. doi: 10.1161/CIRCGEN.121.003535 36170352 PMC9588629

[10] Gulati M, Khan N, George M, et al. Ischemia with no obstructive coronary artery disease (INOCA): A patient self-report quality of life survey from INOCA international. *Int J Cardiol*. Sep 23 2022; doi: 10.1016/j.ijcard.2022.09.047 36162521

[11] Ullah M, Wahab A, Khan SU, et al. Stent as a Novel Technology for Coronary Artery Disease and their Clinical Manifestation. *Curr Probl Cardiol*. Sep 23 2022:101415. doi: 10.1016/j.cpcardiol.2022.101415 36155199

[12] Yang R, Zhang W, Wang X, et al. Nonlinear association of 1,5-anhydroglucitol with the prevalence and severity of coronary artery disease in chinese patients undergoing coronary angiography. *Front Endocrinol (Lausanne)*. 2022;13:978520. doi: 10.3389/fendo.2022.978520 36133308 PMC9483025

[13] Zhu H, Yin C, Schoepf UJ, et al. Machine Learning for the Prevalence and Severity of Coronary Artery Calcification in Nondialysis Chronic Kidney Disease Patients: A Chinese Large Cohort Study. *J Thorac Imaging*. May 3 2022; doi: 10.1097/RTI.0000000000000657 35576551 PMC9592158

[14] Agrawal S, Klarqvist MDR, Emdin C, et al. Selection of 51 predictors from 13,782 candidate multimodal features using machine learning improves coronary artery disease prediction. *Patterns (N Y)*. Dec 10 2021;2(12):100364. doi: 10.1016/j.patter.2021.100364 34950898 PMC8672148

[15] Akella A, Akella S. Machine learning algorithms for predicting coronary artery disease: efforts toward an open source solution. *Future Sci OA*. Mar 29 2021;7(6):FSO698. doi: 10.2144/fsoa-2020-0206 34046201 PMC8147740

[16] Al’Aref SJ, Maliakal G, Singh G, et al. Machine learning of clinical variables and coronary artery calcium scoring for the prediction of obstructive coronary artery disease on coronary computed tomography angiography: analysis from the CONFIRM registry. *Eur Heart J*. Jan 14 2020;41(3):359–367. doi: 10.1093/eurheartj/ehz565 31513271 PMC7849944

[17] Alizadehsani R, Abdar M, Roshanzamir M, et al. Machine learning-based coronary artery disease diagnosis: A comprehensive review. *Comput Biol Med*. Aug 2019;111:103346. doi: 10.1016/j.compbiomed.2019.103346 31288140

[18] Deka P, Blesa J, Pathak D, et al. Combined Dietary Education and High-Intensity Interval Resistance Training Improve Health Outcomes in Patients with Coronary Artery Disease. *Int J Environ Res Public Health*. Sep 10 2022;19(18) doi: 10.3390/ijerph191811402 36141673 PMC9517078

[19] Lee YH, Tsai TH, Chen JH, et al. Machine learning of treadmill exercise test to improve selection for testing for coronary artery disease. *Atherosclerosis*. Jan 2022;340:23–27. doi: 10.1016/j.atherosclerosis.2021.11.028 34871817

[20] Vandeloo B, Andreini D, Brouwers S, et al. Diagnostic performance of exercise stress tests for detection of epicardial and microvascular coronary artery disease: the UZ Clear study. *EuroIntervention*. Sep 23 2022; doi: 10.4244/EIJ-D-22-00270 36147027 PMC9909457

[21] Zaccardi F, Timmins IR, Goldney J, et al. Self-reported walking pace, polygenic risk scores and risk of coronary artery disease in UK biobank. *Nutr Metab Cardiovasc Dis*. Sep 2 2022; doi: 10.1016/j.numecd.2022.08.021 36163213

[22] Mekhael M, Marrouche N, Hajjar AHE, Donnellan E. The Relationship between Atrial Fibrillation and Coronary Artery Disease: Understanding common denominators. *Trends Cardiovasc Med*. Sep 28 2022; doi: 10.1016/j.tcm.2022.09.006 36182022

[23] Peksa JW, Pawlik A, Dziewierz A, Zawislak B. Unexpected severe coronary artery disease in a young patient with only one modifiable risk factor. *Kardiol Pol*. Sep 23 2022; doi: 10.33963/KP.a2022.0224 36148913

[24] Peng Y, Cheng Z, Yi Q. A practical nomogram for predicting coronary thrombosis for Kawasaki disease patients with medium or large coronary artery aneurysm. *Clin Exp Med*. Sep 23 2022; doi: 10.1007/s10238-022-00893-2 36151486

[25] Chen W, Shi S, Jiang Y, et al. Association of sarcopenia with ideal cardiovascular health metrics among US adults: a cross-sectional study of NHANES data from 2011 to 2018. *BMJ Open*. Sep 23 2022;12(9):e061789. doi: 10.1136/bmjopen-2022-061789 36153025 PMC9511583

[26] QuickStats: Percentage* of Adults Aged ≥18 Years with Diagnosed Heart Disease,(dagger) by Urbanization Level (section sign) and Age Group—National Health Interview Survey, United States, 2020 (paragraph sign). *MMWR Morb Mortal Wkly Rep*. Jun 10 2022;71(23):778. doi: 10.15585/mmwr.mm7123a4 35679171 PMC9181055

[27] Al-Shoaibi AAA, Li Y, Song Z, et al. Association of Low-Density Lipoprotein Cholesterol with Risk of Coronary Heart Disease and Stroke among Middle-Aged Japanese Workers: An Analysis using Inverse Probability Weighting. *J Atheroscler Thromb*. Jul 13 2022; doi: 10.5551/jat.63519 35831131 PMC10164596

[28] Caselli C, De Caterina R, Smit JM, et al. Triglycerides and low HDL cholesterol predict coronary heart disease risk in patients with stable angina. *Sci Rep*. Oct 20 2021;11(1):20714. doi: 10.1038/s41598-021-00020-3 34671067 PMC8528835

[29] Chen BW, Liu JJ, Xing JH, et al. Analysis of the Correlation Between the Ratio of Monocytes to High-Density Lipoprotein Cholesterol and in-Stent Restenosis in Patients with Premature Coronary Heart Disease. *Clin Appl Thromb Hemost*. Jan-Dec 2022;28:10760296221079334. doi: 10.1177/10760296221079334 35187964 PMC8864282

[30] Cheng Q, Li Z, Wang X, et al. Relation Between New York Heart Association Functional Class and Remnant Cholesterol, and Non-high Density Lipoprotein Cholesterol in Coronary Heart Disease Patients With Type 2 Diabetes Mellitus. *Angiology*. Apr 24 2022:33197221091315. doi: 10.1177/00033197221091315 35466706

[31] Doi T, Langsted A, Nordestgaard BG. Elevated Remnant Cholesterol Reclassifies Risk of Ischemic Heart Disease and Myocardial Infarction. *J Am Coll Cardiol*. Jun 21 2022;79(24):2383–2397. doi: 10.1016/j.jacc.2022.03.384 35710189 PMC8972554

[32] Ghanavati M, Alipour Parsa S, Nasrollahzadeh J. A calorie-restricted diet with nuts favourably raises plasma high-density lipoprotein-cholesterol in overweight and obese patients with stable coronary heart disease: A randomised controlled trial. *Int J Clin Pract*. Sep 2021;75(9):e14431. doi: 10.1111/ijcp.14431 34080258

[33] Guan J, Wu L, Xiao Q, Pan L. Levels and clinical significance of serum homocysteine (Hcy), high-density lipoprotein cholesterol (HDL-C), vaspin, and visfatin in elderly patients with different types of coronary heart disease. *Ann Palliat Med*. May 2021;10(5):5679–5686. doi: 10.21037/apm-21-1001 34107717

[34] Hayajneh AA, Alhusban IM, Rababa M. The Role of Traditional Obesity Parameters in Predicting Frailty among Coronary Artery Disease Patients Undergoing Cardiac Catheterization. *Int J Clin Pract*. 2022;2022:8676274. doi: 10.1155/2022/8676274 36160288 PMC9484977

[35] Hu Z, Cui J, Li X, Zhou Y, Cai L, Zhang S. High-Density Lipoprotein Cholesterol in Young Nondiabetic Coronary Heart Disease Patients. *Cardiol Res Pract*. 2021;2021:2970568. doi: 10.1155/2021/2970568 34336270 PMC8292093

[36] Jaishankar T, Shivasekar M, Vinodhini VM. Assessment of Remnant Lipoprotein Cholesterol and Oxidized Low density Lipoprotein Associated with Low-grade Inflammation in Coronary Heart Disease Subjects of Young South Indian Population. *J Assoc Physicians India*. Jun 2022;70(6):11–12. doi: 10.5005/japi-11001-0009 35702834

[37] Kuusisto S, Karjalainen MK, Tillin T, et al. Genetic and observational evidence: No independent role for cholesterol efflux over static high-density lipoprotein concentration measures in coronary heart disease risk assessment. *J Intern Med*. Jul 2022;292(1):146–153. doi: 10.1111/joim.13479 35289444 PMC9311699

[38] Liu B, Fang L, Xiong Y, et al. A Machine Learning Model Based on Genetic and Traditional Cardiovascular Risk Factors to Predict Premature Coronary Artery Disease. *Front Biosci (Landmark Ed)*. Jul 4 2022;27(7):211. doi: 10.31083/j.fbl2707211 35866398

[39] Manduchi E, Le TT, Fu W, Moore JH. Genetic Analysis of Coronary Artery Disease Using Tree-Based Automated Machine Learning Informed By Biology-Based Feature Selection. *IEEE/ACM Trans Comput Biol Bioinform*. May-Jun 2022;19(3):1379–1386. doi: 10.1109/TCBB.2021.3099068 34310318 PMC9291719

[40] Orlenko A, Kofink D, Lyytikainen LP, et al. Model selection for metabolomics: predicting diagnosis of coronary artery disease using automated machine learning. *Bioinformatics*. Mar 1 2020;36(6):1772–1778. doi: 10.1093/bioinformatics/btz796 31702773 PMC7703753

[41] Dogdus M, Dindas F, Yenercag M, et al. The Role of Systemic Immune Inflammation Index for Predicting Saphenous Vein Graft Disease in Patients with Coronary Artery Bypass Grafting. *Angiology*. Sep 25 2022:33197221129356. doi: 10.1177/00033197221129356 36154493

[42] Han M, Sun Y, Li N. Relationship between platelet-to-lymphocyte ratio and Coronary Artery Lesion in non-diabetic patients with coronary heart disease. *J Pak Med Assoc*. Jul 2022;72(7):1426–1428. doi: 10.47391/JPMA.3277 36156573

[43] Saint Croix G, Lacy SC, Gazzhal A, et al. Dual Antiplatelet Therapy in Patients Aged 75 Years and Older with Coronary Artery Disease: A Meta-Analysis and Systematic Review. *J Interv Cardiol*. 2022;2022:3111840. doi: 10.1155/2022/3111840 36176329 PMC9499790

[44] Cheang I, Zhu X, Zhu Q, et al. Inverse association between blood ethylene oxide levels and obesity in the general population: NHANES 2013–2016. *Front Endocrinol (Lausanne)*. 2022;13:926971. doi: 10.3389/fendo.2022.926971 36171904 PMC9510609

[45] Guo X, Li N, Wang H, et al. Exploratory analysis of the association between pyrethroid exposure and rheumatoid arthritis among US adults: 2007–2014 data analysis from the National Health and Nutrition Examination Survey (NHANES). *Environ Sci Pollut Res Int*. Sep 24 2022; doi: 10.1007/s11356-022-23145-y 36151437

[46] Wang X, Xiao P, Wang R, et al. Relationships between urinary metals concentrations and cognitive performance among U.S. older people in NHANES 2011–2014. *Front Public Health*. 2022;10:985127. doi: 10.3389/fpubh.2022.985127 36148349 PMC9485476

