# Supplementary material for: Use of machine learning to identify risk factors for coronary artery disease
Source: PLoS One. 2023 Apr 14;18(4):e0284103. doi: 10.1371/journal.pone.0284103 (PMC10104376; doi:10.1371/journal.pone.0284103)
Supplement: Attachment — Submitted filename: reviewer rebuttle.docx [file pone.0284103.s001.docx]

The manuscript must describe a technically sound piece of scientific research with data that supports the conclusions. Experiments must have been conducted rigorously, with appropriate controls, replication, and sample sizes. The conclusions must be drawn appropriately based on the data presented.

Reviewer #1: Yes

Reviewer #2: Yes

2. Has the statistical analysis been performed appropriately and rigorously?

Reviewer #1: Yes

Reviewer #2: Yes

3. Have the authors made all data underlying the findings in their manuscript fully available?

The [PLOS Data policy](https://urldefense.com/v3/__http:/www.plosone.org/static/policies.action*sharing__;Iw!!Dq0X2DkFhyF93HkjWTBQKhk!VWAulOrAqVqF-v1uCf5kOvpB9Ojswkg2fc6KJYqhLPHuN6_sEX6Tt0E5Rqoi2-z6kx5lnYoUqCX25y_R1iLkpCtbuGOO$) requires authors to make all data underlying the findings described in their manuscript fully available without restriction, with rare exception (please refer to the Data Availability Statement in the manuscript PDF file). The data should be provided as part of the manuscript or its supporting information, or deposited to a public repository. For example, in addition to summary statistics, the data points behind means, medians and variance measures should be available. If there are restrictions on publicly sharing data—e.g. participant privacy or use of data from a third party—those must be specified.

Reviewer #1: Yes

Reviewer #2: Yes

4. Is the manuscript presented in an intelligible fashion and written in standard English?

PLOS ONE does not copyedit accepted manuscripts, so the language in submitted articles must be clear, correct, and unambiguous. Any typographical or grammatical errors should be corrected at revision, so please note any specific errors here.

Reviewer #1: Yes

Reviewer #2: Yes

5. Review Comments to the Author

Please use the space provided to explain your answers to the questions above. You may also include additional comments for the author, including concerns about dual publication, research ethics, or publication ethics. (Please upload your review as an attachment if it exceeds 20,000 characters)

Reviewer #1: General comments

Integrating the Machine-learning model with the usual statistical analysis model can be a promising revolution in e-Health ad m-Health to solve complex issues accurately and effectively.

The crux of this manuscript is the use of new analytical techniques to complement the usual statistical model. The authors have used machine learning to predict the risk of CAD by capturing the actual physiological relationship of the factors associated with the risk of CAD. The manuscript is well written: methodology is well narrated and executed; the results are well presented, and the discussion is well articulated.

The manuscript lacks page numbers; please insert them in the next version of the manuscript.

**###Thank you for your suggestion. The authors have added page numbers.**

Specific comments

Abstract:

- Line 24: The author should define the abbreviation “SHAP.” Most abbreviations must be defined upon first use unless otherwise explained.

**### Thank you for your suggestion. The authors have defined the abbreviation SHAP on the first instance of its use.**

Methods:
- Line 91: Why p<0.0001 was the cut-off level of statistical significance to include covariate in the machine-learning model? Is there any reference or statistical explanation?
\##**Thank you for your inquiry.** A p-value of 0.05 is commonly used. Since we had 684 covariates – we adjusted for multiple testing with a Bonferroni adjustment, the most conservative methodology of adjustment. Then we rounded to the nearest value.

Discussion:
- Line 200: Misspelled word - Replace “contrary” artery disease with “coronary” artery disease.

**### Thank you for your suggestion. The authors have made the edit.**

Reviewer #2: Thanks very much for this very interesting and informative article. I feel that overall the paper was well written and without significant flaws. The study objectives and the methods section are clearly defined, the article is easily readable, and the topic is relevant to the readership. Limitations are adequately addressed. Conclusions are appropriate for the scope of the study. The paper is formally correct and it is clear its clinical relevance, and what this article should add to the body of knowledge on this topic.

**### Thank you for your positive feedback.**
